# Micromagnetic Simulations of Anisotropies in Coupled and Uncoupled Ferromagnetic Nanowire Systems

**DOI:** 10.1155/2013/472597

**Published:** 2013-10-09

**Authors:** T. Blachowicz, A. Ehrmann

**Affiliations:** ^1^Institute of Physics, Center for Science and Education, Silesian University of Technology, Krzywoustego 2, 44100 Gliwice, Poland; ^2^Faculty of Textile and Clothing Technology, Niederrhein University of Applied Sciences, 41065 Mönchengladbach, Germany

## Abstract

The influence of a variation of spatial relative orientations onto the coupling dynamics and subsequent magnetic anisotropies was modeled in ferromagnetic nanowires. The wires were analyzed in the most elementary configurations, thus, arranged in pairs perpendicular to each other, leading to one-dimensional (linear) and zero-dimensional (point-like) coupling. Different distances within each elementary pair of wires and between the pairs give rise to varying interactions between parallel and perpendicular wires, respectively. Simulated coercivities show an exchange of easy and hard axes for systems with different couplings. Additionally, two of the systems exhibit a unique switching behavior which can be utilized for developing new functionalities.

## 1. Introduction 

Innovative magnetic storage and sensing devices, magnetic logical circuits (MLC), magnetic quantum cellular automata (MQCA), and other novel magnetic systems are based on nanoscale ferromagnetic structures [1–7]. Magnetic states of reduced dimensionality appearing in patterned nanostructures enable achievement of much higher areal densities than in conventional media for data-storage applications [8]. Additionally, like in MLCs, they allow for pure magnetic signal processing without the use of an electron current flow and subsequently lowered energy consumption.

For patterned magnetic structures [9–13], it is important to enable a reliable coupling between neighboring magnetic units ordered in a set for consecutive dynamic operations. For coupled nanorings, for example, the overlap of neighboring rings has shown a strong influence on the signal transport properties in logical NOT gates, and so forth. [6], while in some systems a nonnegligible probability of unintentional signal inversions due to random effects was observed [14, 15]. Thus, the examination of cooperative processes in magnetic highly-ordered arrays of nanowires is of utmost importance for understanding steady-states and, even more important, for understanding basic functionalities and requirements to realize magnetization dynamics in nanochains and nanowires.

Recent progresses in technology and measurement techniques of ferromagnetic nanowires give opportunities to analyze novel effects and applications [16–19]. While the behavior of single ferromagnetic wires is well understood, simply governed by the shape anisotropy, the interplay between spatially ordered nanowires of different orientations and coupling requires elementary studies [20, 21]. Since most commercial magnetic solutions nowadays are based on thin-layers technology, where the coupling between different materials can tailor these devices' functionalities via surface or bulk interactions (2D or 3D; surface or bulk), we analyze an approach which uses coupling between parallel wires (1D coupling) and/or perpendicularly oriented ones (0D coupling). The results of magnetization dynamics point out new functionalities of these novel wire sets. In this way, the results presented here broaden the knowledge about shape, size, and composition analysis of nanowire magnetism [22, 23]. 

## 2. Materials and Methods

**Figure 1:**
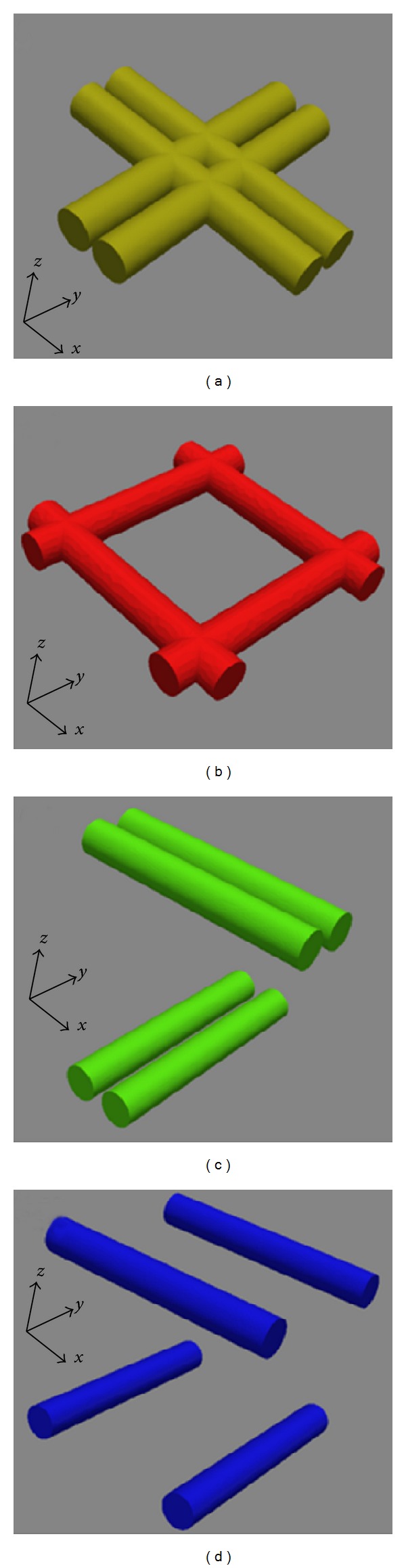
Magnetic systems composed of four nanowires, arranged in perpendicular pairs, with a different coupling within one pair/between the pairs: maximum coupling between the pairs and (a) coupling/(b) no coupling within each pair; no coupling between the pairs and (c) coupling/(d) no coupling within each pair.

Systems of four iron (Fe) wires with diameter 10 nm and length 70 nm, ordered in four different configurations (Figure 1), have been modeled using the finite element method. This order of magnitude is comparable with recent experimental results of patterns produced by interference lithography [24, 25]. The orientations of the wires in one plane or in two planes above each other have been chosen since such systems can be produced by photo-lithography, either in one step or in form of three layers with nonmagnetic materials between the magnetic wires. Additionally, the nontouching wires can serve as model systems for magnetic nanowires which are laid down on each other, either touching each other or with a certain distance due to a nonmagnetic cover over the magnetic core.

The “Parallel Finite Element Micromagnetics Package (MAGPAR)” [26] has been used for simulation, working with the Landau-Lifshitz-Gilbert (LLG) equation of motion $\partial \vec{M}/\partial t=-(\gamma /\left(1+\alpha^{2})\right)\vec{M}\times {\vec{H}}_{\text{eff}}-(\gamma \alpha /\left(1+\alpha^{2}\right)M_{s})\vec{M}\times \left(\vec{M}\times {\vec{H}}_{\text{eff}}\right)$ with the magnetization $\vec{M}$, the saturation magnetization *M*_*s*_, the effective field ${\vec{H}}_{\text{eff}}$, the gyromagnetic ratio *γ*, and a dimensionless damping constant *α*.

For meshing, finite tetrahedral elements of dimensions of maximal 3 nm were used. This value is significantly smaller than the Fe exchange length which exceeds 20 nm [27]. The other physical parameters were exchange constant *A* = 2 · 10^−11^ J/m, magnetic polarization at saturation *J*_*s*_ = 2.1 T, and the Gilbert damping constant *α* = 0.01 [28]. These values are typically used for iron samples.

For the simulations, four different systems with the following coupling possibilities were taken into account (Figure 1): exchange dominated coupling between directly crossed pairs of wires (a), direct coupling at wire ends with other coupling mechanisms (dipolar, exchange) omitted (b), exchange dominated coupling within each pair and no magnetic coupling between the pairs (c), and a case of four wires with all magnetic coupling contributions excluded, while the wires are placed at infinite distance to each other (d).

Simulations were carried out with the external magnetic field applied in the sample plane, for a sample orientation of 0° along the *x*-axis which is always parallel to one pair of wires. Beginning at a random magnetization state (i.e., external field *H*_ext_ = 0), the field was changed at a constant speed of 10 kA/(m·ns) up to 800 kA/m to saturate the sample. Afterwards, the field was swept at the same speed to −800 kA/m to obtain negative saturation, and afterwards back to positive saturation again. The field sweeping speed is comparable to typical values in MRAM applications [29]. The test with slower sweep rates did not show significant differences in results. 

## 3. Results and Discussion

**Figure 2:**
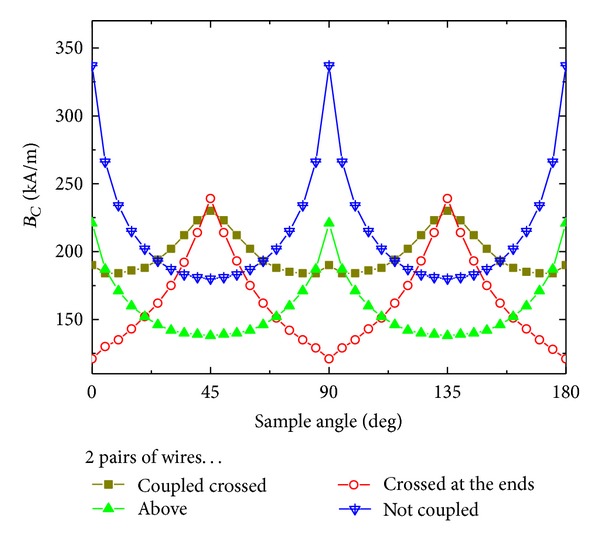
Simulated coercivities of the magnetic systems depicted in Figure 1.

The coercivities *B*_*C*_ of these four magnetic nanosystems have been derived from the simulated hysteresis loops.  Figure 2 shows the calculated values of *B*_*C*_ for the angular region of 0° ⋯ 180°. As expected in systems with fourfold geometry, a fourfold anisotropy can be detected in all four situations. From former simulations [30] it is known that a “typical” fourfold anisotropy leads to sharp maxima of *B*_*C*_ along the easy axes and broad minima around the hard axes, as can be seen here for situations (c) and (d), that is, no coupling between the two pairs. However, both the other situations with coupling between the two pairs show deviations from this behavior. Assuming that the identification of easy axes by maxima in the coercivity is universal and can thus be used for all cases under examination, even an exchange of easy and hard axes of the systems, resulting from different couplings between the pairs of nanowires, can be recognized. Apparently this finding can be attributed to the direct ferromagnetic exchange coupling, which is dominant in cases (a) and (b), opposite to systems (c) and (d) where this coupling does not play a role. 

**Figure 3:**
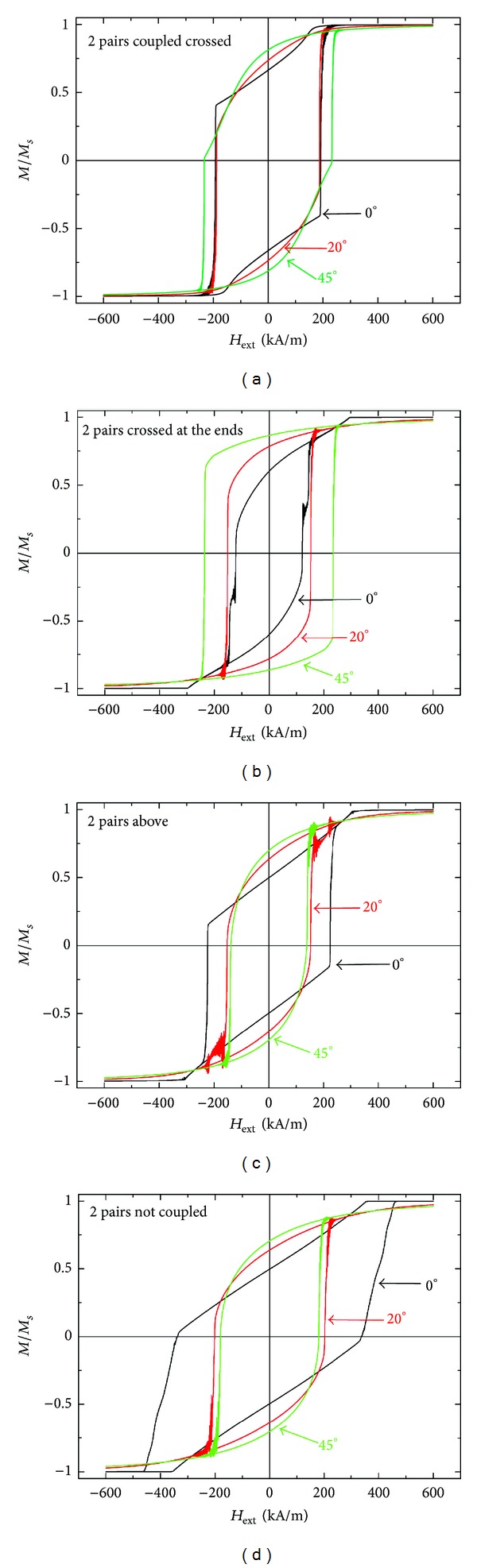
Simulated hysteresis curves for the samples depicted in Figure 1, exemplarily shown for orientations relative to the external magnetic field of 0° (i.e., parallel to one pair of wires), 20°, and 45°.

For a deeper understanding of this finding, the simulated hysteresis loops are depicted in Figure 3 for orientations relative to the external magnetic field of 0° (i.e., parallel to one pair of wires), 20°, and 45°. In all four cases, the simulated loops show deviations from “typical” hysteresis curves. Mostly, the shapes can be regarded as composed of two hysteresis loops, one with typical attributes of an easy axis (i.e., broad loop with abrupt magnetization changes) and one with typical signs of a hard direction (i.e., narrow loop with broad transition regions from one saturated state to the other). Apparently, easy and hard axes cannot easily be defined in the wire systems under examination. Case (d), where no coupling between the wires exists, corresponds to a pure superposition of the individual wires, while the coupled cases (a) and (b) show completely different hysteresis loops. 

Comparing Figure 2 with absolute maxima in the coercivity around 0° for cases (c) and (d) and Figure 3 with abrupt magnetization changes at 0° for cases (a) and (c), it is obvious that the identification of an easy fourfold axis by a maximum in the coercivity is no longer valid in the systems under examination. Instead, the coercivities are related to the magnetization reversal processes which may differ in dependence of the coupling configuration and the angle.

A detailed study of magnetization reversal processes in fourfold nanowire systems similar to sample (b) of different dimensions has revealed the existence of six different reversal mechanisms, depending on wire length and diameter as well as on the orientation of the system to the external magnetic field (to be published). Additionally, for sample (b) even a step in the hysteresis loop can be found, which has been previously reported as a typical feature of some exchange bias systems [31, 32]. This finding supports the idea of exploring differently spaced magnetic nanowires to examine the influence of variable dipole coupling fields on the magnetic properties of such systems—not only to understand the mechanisms better but also to find systems with novel interesting and unexpected properties (e.g., [33]).

## 4. Conclusion

In conclusion, our micromagnetic simulations of systems consisting of two perpendicular pairs of parallel wires—as an alternative to exchange and/or dipolar coupled layered systems—have shown the strong dependence of the magnetic properties on the kind of coupling between them. Additionally, some systems exhibit unexpected features which are promising for the development of new functionalities.
